# COVID-19 Disease and Dermatomyositis: A Mini-Review

**DOI:** 10.3389/fimmu.2021.747116

**Published:** 2022-01-13

**Authors:** Jie Qian, Hui Xu

**Affiliations:** Department of Rheumatology, Affiliated Hospital of Nantong University, Nantong, China

**Keywords:** SARS-CoV-2, COVID-19, dermatomyositis, pathogenesis, treatment

## Abstract

The pandemic of coronavirus disease 2019 (COVID-19) caused by SARS-CoV-2 has caused a large number of deaths, and there is still no effective treatment. COVID-19 can induce a systemic inflammatory response, and its clinical manifestations are diverse. Recently, it has been reported that COVID-19 patients may develop myositis and interstitial pulmonary disease similar to dermatomyositis (DM). This condition is similar to the rapidly progressive interstitial lung disease associated with MDA5^+^ DM that has a poor prognosis and high mortality, and this poses a challenge for an early identification. Suppression of the immune system can protect COVID-19 patients by preventing the production of inflammatory cytokines. This article attempts to explore the possibility of a relationship between COVID-19 and DM in terms of the potential pathogenesis and clinical features and to analyze the therapeutic effect of the immunosuppressive drugs that are commonly used for the treatment of both DM and COVID-19.

## Introduction

In December 2019, a novel infectious disease caused by severe acute respiratory syndrome coronavirus 2 (SARS-CoV-2) was reported (1). Compared with SARS-CoV and MERS-CoV, SARS-CoV-2 is more infectious; therefore, coronavirus disease 2019 (COVID-19) has become a global epidemic. The most common symptoms of COVID-19 are fever, cough, dyspnea, myalgia, and fatigue (2), and pulmonary involvement is significantly associated with a poor prognosis and a high mortality. Viruses can cause a variety of autoimmune diseases, and patients with autoimmune diseases are more susceptible to infection due to the pathogenesis of the autoimmune disease and/or the use of immunosuppressants. Among the potential autoimmune diseases that may be related to COVID-19, the most intriguing is idiopathic inflammatory myopathy (IIM), a heterogeneous disease that primarily affects the skeletal muscles and can be divided into polymyositis, dermatomyositis (DM), immune-mediated necrotizing myopathy, anti-synthase syndrome, and inclusion body myositis (3). During the COVID-19 outbreak, several studies have recognized a striking similarity between COVID-19 and DM due to the lung and muscle involvement and the presence of a rash. DM typically presents with characteristic skin manifestations that accompany or precede muscle weakness, and interstitial lung disease (ILD) is a common complication of DM. The pathogenesis of DM is still unclear, although it is likely associated with inappropriate complement activation and interferon (IFN) response (4, 5), which results in the production of myositis-specific autoantibodies that may be related to unique clinical features. Obviously, COVID-19 is closely related to DM.

## COVID-19 and Autoimmunity

For many infections, the immune system provides an appropriate response that mediates resistance to the invading microorganism. Appropriate innate and adaptive responses promote the coordinated production of proinflammatory cytokines that can control pathogens. Several environmental factors have been suspected to trigger or even exacerbate existing autoimmune conditions in genetically susceptible patients, and these factors include viral, bacterial, and parasitic infections (6). Viruses can induce autoimmunity in genetically susceptible individuals through multiple mechanisms, including molecular mimicry, epitope spreading, bystander activation, and the immortalization of infected B cells. Chronic relapsing/reactivated infections caused by Epstein-Barr virus (EBV) are associated with the occurrence or onset of various autoimmune diseases, including DM (7). Similar to SARS-CoV, SARS-CoV-2 uses the membranous angiotensin-converting enzyme 2 (ACE2) receptors to enter human cells (8). Then, SARS-CoV-2 can trigger innate and adaptive immune responses, known as cytokine storms. This abnormal elevation of inflammatory cytokines, such as interleukin (IL)-1, IL-6, IL-10, interferon γ (IFN-γ), monocyte chemotactic protein-1 (MCP-1), and granulocyte-macrophage colony-stimulating factor, can interact with the complement and coagulation systems, leading to acute respiratory distress syndrome, disseminated intravascular coagulation, and even multiple organ failure (9). Li et al. demonstrated significant expression of ACE2 receptors on alveolar epithelial cells, which may explain why pulmonary involvement is a hallmark of COVID-19 (10). A retrospective study revealed that SARS-CoV-2 might act primarily on lymphocytes, especially T lymphocytes. Moreover, patients with severe COVID-19 had higher neutrophils and fewer lymphocytes than patients with mild COVID-19, indicating that an increased neutrophil-to-lymphocyte ratio (NLR) in patients with severe COVID-19 and the use of lymphocyte subset monitoring are helpful for early screening, diagnosis, and treatment of severe COVID-19 (11).

## Pathogenesis and Clinical Characteristics of DM

DM is a rare disease characterized by distinct cutaneous manifestations and a clinically heterogeneous systemic presentation. Pathognomonic skin manifestations such as Gottron’s papules and heliotrope rash are conducive to the early diagnosis of DM. Therefore, DM can be difficult to diagnose in the absence of the characteristic dermatologic features. DM has been suggested to occur more frequently in women and African Americans (12), and the age range of the patients at the time of the diagnosis of DM is bimodal, with the peak frequencies occurring in 5- to 14-year-old children and in 40- to 60-year-old adults (13). Epidemiological data has demonstrated that subsets of DM patients that were diagnosed by the presence of different myositis-specific autoantibodies tend to be diagnosed at specific times of the year (14). The pathogeneses of the different types and subtypes of myositis are incompletely understood. Predisposition to IIM are attributable to genetic and environmental factors, and viral or bacterial infections may induce the occurrence of the disease. The seasonal clustering of symptoms suggests that viral or bacterial infections could be common environmental triggers. A case-control study showed that a higher number EBV genomes are present and that anti-Epstein-Barr nuclear antigen 1 antibodies were detected at a higher frequency in DM patients than in healthy control counterparts (15). Autoimmune mechanisms are important in the pathogenesis of IIM, and myositis-specific or associated autoantibodies can be observed in patients with IIM, which is often associated with particular clinical features (16). With the further study of DM, many novel autoantibodies against myositis have been found, including anti-melanoma differentiation-associated gene 5 (MDA5), anti-transcriptional intermediary factor 1 γ, anti-nuclear matrix protein 2, anti-Ku and anti-Mi-2 (17). Anti-MDA5^+^ DM is typically associated with rapidly progressive interstitial lung disease (RP-ILD), which has a high early mortality (18). It is still controversial whether the immune activation seen in DM is antibody-dependent or is triggered by a classical complement cascade (4). The IFN pathway is activated in the different clinical subtypes of myositis, and type 1 IFN1 is most upregulated in DM patients (5). The expression level of IFN-induced genes was correlated with the indicators of DM disease activity (19). Three different ligand families activate the IFN pathway by binding to cell surface receptors and by stimulating the expression of IFN-inducible genes *via* the Janus kinase (JAK)/signal transducer and the activator of transcription (STAT) signaling pathway (20). A persistent IFN response promotes antigen presentation and lymphocyte responses and induces chemokine expression, and the resulting T and B cell activation may also be responsible for the production of autoantibodies (20).

Approximately 80% of DM patients have myopathy, which usually presents as proximal muscle weakness (4). Extramuscular involvement is common in IIM; the skin, lungs, joints, heart, and gastrointestinal tract can be affected in IIM; and the degree of involvement varies according to the different subtypes of IIM (21). ILD is a common complication of DM, and the severity of the pulmonary symptoms varies among patients with different subtypes (22). Patients with mild ILD are stable and respond well to treatment, while some patients with RP-ILD have more severe disease and have a poor prognosis (23, 24). In addition, the extent of muscle involvement can also be fatal for DM patients because weakness of the intercostal muscles may lead to respiratory disorders and acute respiratory distress syndrome (25).

## Correlation on COVID-19 and DM

**Figure 1:**
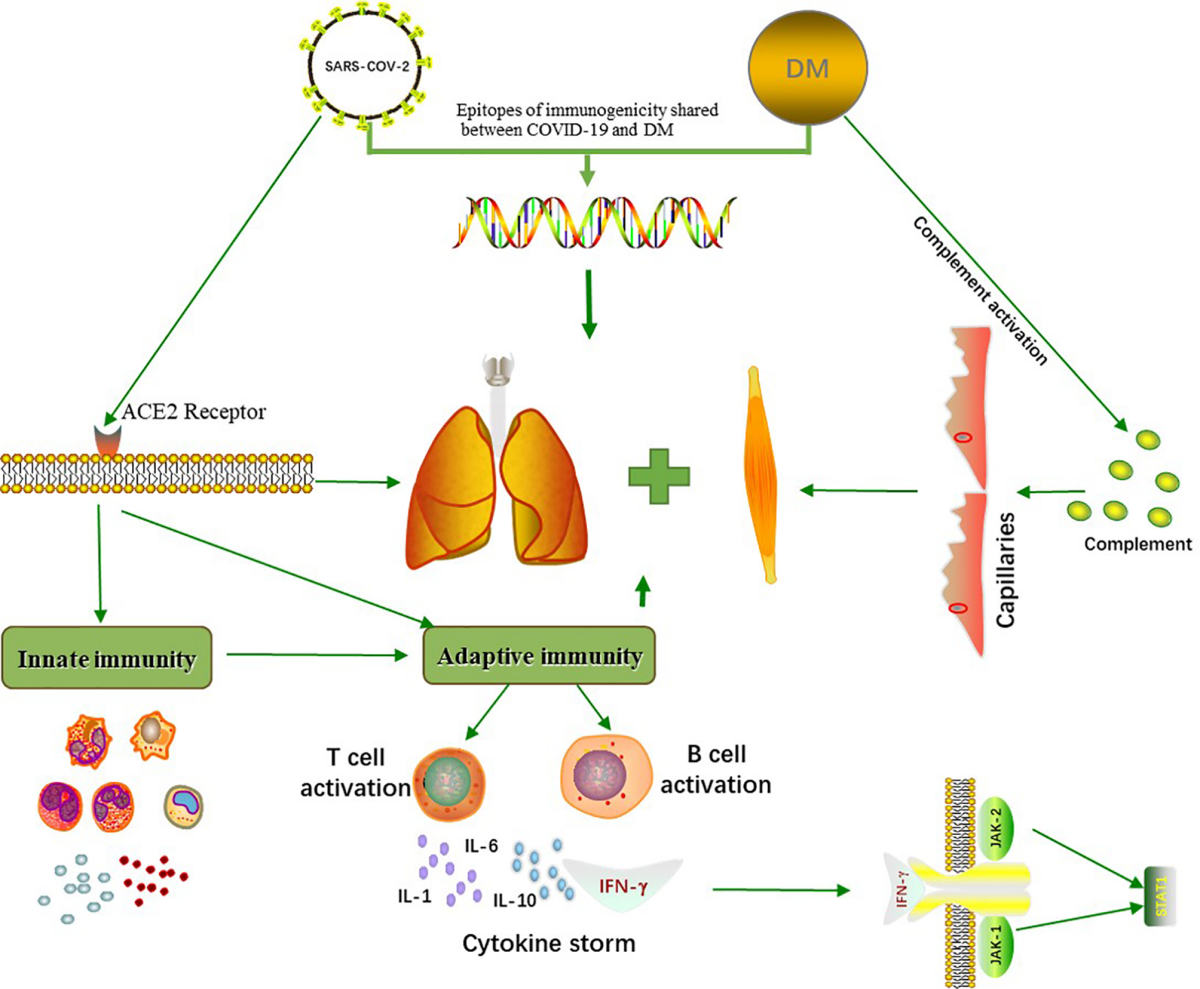
Common pathogenetic mechanisms between COVID-19 and DM. COVID-19 and DM share three immunogenic linear epitopes with high sequence consistency. SARS-CoV-2 enters human cells through the membranous angiotensin-converting enzyme 2 (ACE2) receptors, triggering an innate and adaptive immune response. This initiates the production of cytokines such as IL-1, IL-6, IL-10 and IFN-γ, which can induce lung and muscle damage. The activation of complement in DM patients results in capillary destruction, which further affects multiple organs, and cytokine storms are associated with the occurrence of certain subtypes of DM.

The similarity of COVID-19 and DM implies a common underlying mechanism (**Figure 1**). MDA5, a myositis-specific autoantibody, is an intracellular sensor of the intermediates or byproducts of double-stranded RNA viral replication that trigger the innate response and subsequent production of cytokines that activate macrophages and helper T cells (26). Increased levels of several cytokines, such as IFN-γ, IL-8, and MCP-1 (which are similar to the cytokine storms seen in severe COVID-19 patients), were found in patients with anti-MDA5^+^ DM (27). IFN1, which plays a major role in the muscle fiber damage in DM patients, is involved in the organ damage seen in COVID-19 (28). Megremis et al. proposed that DM patients have three linear epitopes of immunogenicity that have a high sequence identity with the SARS-CoV-2 protein; thus, a potential exposure to a virus within the coronavirus family may lead to the development of DM (29). An Italian patient study reported a higher prevalence of COVID-19 in patients with autoimmune diseases than in the general population (30). Movahedi et al. noticed a sudden outbreak of new cases of juvenile dermatomyositis during the COVID-19 pandemic, with a higher incidence among women (31). Notably, 76.5% of COVID-19 patients are women (30), and the ratio of males to females in DM is 1:2 (4). Autoimmune diseases are generally more prevalent in women than men, with the strongest sex biases seen in Sjogren’s syndrome, systemic lupus erythematosus, autoimmune thyroid disease, and scleroderma (32). Estrogen, especially 17-β estradiol and prolactin, can act as humoral immune enhancers, which causes women to be more susceptible to autoimmune diseases (33). In addition, because of incomplete X chromosome inactivation caused by random silencing in females during the early stages of embryogenesis, several immune-related genes may be upregulated, and their overexpression may influence the immune response (34, 35). Several cases of DM associated with COVID-19 have been summarized (**Table 1**). Of the seven patients that were reported to have both DM and COVID-19, 85.7% were female, and two of these patients were anti-MDA5^+^. Most patients had distinct cutaneous manifestations, with elevated CK. In addition, the female mortality was 33.3%, even after receiving aggressive treatment. Based on our summary table, we should raise awareness of the fact that women are more susceptible to COVID-19 combined with DM and experience more severe conditions, and effective treatment interventions would help to improve survival. It is increasingly recognized that the features of severe COVID-19 are similar to those of DM, especially anti-MDA5^+^ DM because these conditions have similar involvement of the lung and are associated with the development of rash, fatigue, and myalgia. In addition, the imaging findings of COVID-19 are comparable to those of the ILD seen in anti-MDA5^+^ DM because diffuse ground-glass opacifications are often present in both of these diseases, and the presence of the ground-glass opacifications suggest peribronchovascular consolidation (26). Compared with other DM subtypes, the RP-ILD seen in anti-MDA5^+^ DM usually develops rapidly, rarely relapses, and responds poorly to glucocorticoids and immunosuppressants, which seems to be similar to a viral infection (27). In recent case reports, autoantibodies against myositis, such as anti-MDA5, anti-SAE, anti-Mi2, anti-Ku, and anti-Ro52, have been detected in COVID-19 patients with DM. Viral infections seem to be associated with certain subtypes of DM.

**Table 1:** Cases of COVID-19-associated DM.

Authors	Year	Sex	Age	Antibody	Clinical characteristics	Treatment	Outcomes
Zhang et al. (36)	2020	F	58	Anti-SSA, Anti-SAE, Anti-Ku	Muscle weakness, dyspnea, myalgia, CK↑	Antiviral drug, MP, HCQ, tocilizumab	Recovered
Kogami et al. (37)	2020	F	46	Anti-MDA5	Erythematous papules	Antiviral drug	Recovered
Cao et al. (38)	2020	F	45	Anti-Ro52	Fever, myalgia, Gottron’s sign, RP-ILD, ferritin↑, IL-6↑, CK↑, LDH↑	Antiviral drug, MP, CTX, IVIG	Died
Borges et al. (39)	2021	F	36	Anti-Mi2	Sore throat, fatigue, Gottron’s papules, muscle weakness, Raynaud phenomenon, CK↑	MP	Recovered
Quintana-Ortega et al. (40)	2021	F	11	Anti-MDA5, Anti- Ro/SSA	Gottron’s papules with calcinosis, heliotrope rash, palmar papules, dysphagia, cervical subcutaneous emphysema, RP-ILD, SF↑, IL-6↑	Antiviral drug, glucocorticoids, HCQ, MMF, TAC, CTX, tocilizumab, tofacitinib, IVIG, PE	Died
Ho et al. (41)	2021	M	58	NA	Fatigue, myalgias, muscle weakness, Gottron’s sign, heliotrope rash, CK↑	Glucocorticoids, MTX,	Recovered
Liquidano-Perez et al. (42)	2021	F	4	Anti-RNP/Sm, Anti-Scl-70, Anti-Sm	Muscle weakness, Gottron’s papules, heliotrope rash, dysphagia, CK↑	Glucocorticoids, HCQ, MTX, CsA, IVIG	Recovered

CK, creatine kinase; CTX, cyclophosphamide; CsA, cyclosporine; DM, dermatomyositis; F, female; HCQ, hydroxychloroquine; IL-6, interleukin-6; IVIG, Intravenous immunoglobulin; LDH, lactate dehydrogenase; M, male; MMF, mycophenolatemofetil; MP, methylprednisolone; MTX, methotrexate; NA, not available; PE, plasmapheresis; RP-ILD, rapidly progressive interstitial lung disease; TAC, tacrolimus.

More than 10% of COVID-19 patients were reported to have muscular symptoms and elevated CK levels (43). Muscle biopsies from patients with COVID-19 show abnormal presence of Myxovirus resistance protein A in muscle fibers and capillaries. Myxovirus resistance protein A is one of the IFN1-inducible proteins, is overexpressed in biopsy muscle specimens from DM patients and may be a more sensitive marker of DM (44, 45), suggesting that autoimmune myositis may be caused by COVID-19. The SARS-CoV-2 receptor ACE2 is expressed in a variety of human tissues, including the skeletal muscles, where ACE2 expression is the lowest (10). The expression and distribution of ACE2 remind us that SARS-CoV-2 may cause muscle injury through direct or indirect mechanisms. Beydon et al. reported a case of this type of myositis diagnosed by MRI (46). However, SARS-CoV, which has the same receptor as SARS-CoV-2, was not found in the muscle tissue of the patient at autopsy (47, 48). In addition, the muscle manifestations of COVID-19 patients can be contributed to rhabdomyolysis caused by SARS-CoV-2 (49). Rhabdomyolysis is a life-threatening disease that requires aggressive hydration in order to avoid acute renal failure, which can worsen the oxygenation status in COVID-19 patients (49, 50). Therefore, when patients have focal muscle pain and fatigue, the possibility of rhabdomyolysis should be taken seriously (50). Symptoms of myopathy in severe systemic viral diseases are multifactorial, and further muscle biopsies and antibody screening are required.

## Treatment Options

Since hyperinflammation underlies COVID-19 and is associated with the disease severity, anti-inflammatory therapy may be beneficial to patients. Immune impairment may inhibit viral-induced cytokine storm syndrome (11). Immunosuppressive agents have a variety of mechanisms targeting the cellular and humoral immune responses, so the use of immunosuppressive agents may have a protective effect for COVID-19 patients. Along with the increased understanding of COVID-19, several drugs commonly used to treat DM have potential therapeutic effects for COVID-19 patients.

### Glucocorticoids

Glucocorticoids inhibit inflammatory cytokines and reduce the proliferation and differentiation of lymphocytes and macrophages, and they have immunosuppressive effects on the body and may increase susceptibility to COVID-19 (51). There is no evidence that patients infected with COVID-19 benefit from glucocorticoids, and COVID-19 patients may be more prone to the side effects of glucocorticoid treatment (52). However, retrospective analyses have shown that the use of glucocorticoids reduces hospital mortality in patients with COVID-19 cytokine storms (53). Dexamethasone has been shown to reduce the mortality in patients with COVID-19 who receive invasive mechanical ventilation (54). Strangfeld et al. reported a higher mortality rate in patients receiving higher dosages of glucocorticoids (>10 mg/day prednisolone equivalent dose) than in those who did not receive glucocorticoids (55). The efficacy of glucocorticoids in COVID-19 is controversial, especially in terms of doses and risk of side effects.

### Conventional Synthetic Disease-Modifying Antirheumatic Drugs

Some conventional synthetic disease-modifying antirheumatic drugs (csDMARDs), such as methotrexate, hydroxychloroquine (HCQ), and azathioprine, suppress the cytokine storm associated with COVID-19 (11). Methotrexate, a dihydrofolate reductase inhibitor, is widely used in autoimmune diseases because of its pleiotropic therapeutic effects on various immune cells and mediators and its inhibition of the body’s overall inflammatory response (56). In patients with DM, the use of HCQ can effectively improve the skin lesions. Chloroquine (CQ) blocks the fusion of the virus to the host cell by interfering with the terminal glycosylation of the cell receptor, ACE2, and by inhibiting sialic acid biosynthesis, which is used as a receptor by the viruses through inhibiting quinine reductase-2 (57). On the other hand, CQ can also play an antiviral role by reducing IFN1 (51). Wang et al. found that CQ played an important role in multiple stages of COVID-19 *in vitro*, and it had immunomodulatory effects that synergically enhanced its antiviral effects (58). However, the mortality of COVID-19 patients treated with HCQ increased, and there was no benefit from CQ (59). In addition, adverse drug reactions associated with CQ and HCQ increased dramatically during the COVID-19 pandemic (60). Mycophenolate mofetil, calcineurin inhibitors, and cyclophosphamide as immunosuppressants have also shown a good therapeutic effect in the treatment of myositis. Mycophenolate mofetil is an inhibitor of purine synthesis that inhibits inosine monophosphate dehydrogenase, reduces lymphocyte proliferation, and has antifibrosis properties (61). Mycophenolate mofetil showed anti-SARS-COV-2 activity and it could be worth considering used for clinical treatment of COVID-19 (62). In addition, tacrolimus and cyclosporine produced dose-dependent inhibitory effects on NK cell degranulation and IFN-γ *in vitro* (63), and tacrolimus also reduced the ability of dendritic cells to stimulate T cells, resulting in decreased production of CXCL-10 and IL-12 (64). Tacrolimus has a positive effect on survival in liver transplant patients with symptoms of COVID-19 compared to other immunosuppressants, including cyclosporine and mycophenolate mofetil (65). Nevertheless, there was no improvement in severe COVID-19 patients who received a combination of methylprednisolone pulses and tacrolimus (66). A prospective study demonstrated that a combination of high-dose glucocorticoids, tacrolimus and intravenous cyclophosphamide improved survival in anti-MDA5^+^ DM patients complicated with ILD. This regimen also has an increased risk of opportunistic infections, leading to an exacerbation of ILD (67). Some csDMARDs, such as methotrexate, mycophenolate mofetil, and tacrolimus may provide a therapeutic strategy for COVID-19. The efficacy and safety of CQ and HCQ remain controversial and need further research.

### Interleukin Receptor Antagonists

Considering the mechanism of COVID-19, cytokine-targeting biologicals and signaling molecule inhibitors are also promising therapeutic approaches (11). Anakinra is a recombinant IL-1 receptor antagonist that can be used to treat autoinflammatory diseases (51). Zong et al. demonstrated that patients with myositis may respond to anakinra, especially DM patients with skin rash (68). Currently, a study is underway that combines anakinra with anti-IFN-γ antibodies for the treatment of patients with COVID-19 (51). IL-6 is a pleiotropic cytokine that plays a key role in the cytokine storm. The IL-6 receptor antagonist tocilizumab is used for the treatment of refractory juvenile DM (69). Tocilizumab may be a salvage therapy for anti-MDA5^+^ DM associated with RP-ILD patients refractory to an intensive immunosuppressive regimen (70). A retrospective study of patients with severe COVID-19 showed that tocilizumab improved the clinical symptoms and that the early use of tocilizumab effectively controlled the progression of the symptoms (71). Another study also demonstrated that corticosteroids combined with tocilizumab or anakinra reduced mortality in hospitalized patients with COVID-19 and that corticosteroids combined with tocilizumab had better survival outcomes (53). Interleukin receptor antagonists anakinra and tocilizumab may be alternative drugs for COVID-19 patients, but given the intrinsic limitations of retrospective studies, randomized clinical trials are still warranted.

### JAK Inhibitors

JAK inhibitors are novel target synthetic immunoregulatory agents that not only inhibit tyrosine kinases, which are involved in intracellular viral transport and epithelial endocytosis, but also inhibit intracellular signal transduction of various inflammatory cytokines (51). A patient with refractory JDM was described as having clinical improvement after treatment with baricitinib. The JAK inhibitor baricitinib binds to cyclin G-associated kinases, a regulator of endocytosis, to block the entry of SARS-CoV-2 into cells and the inflammatory cytokine storm (72). A randomized controlled trial demonstrated that baricitinib combined with remdesivir reduced the recovery time in COVID-19 patients, especially in patients receiving high-flow oxygen or noninvasive ventilation, and it also accelerated the clinical improvement of patients and was associated fewer serious adverse events (73). In the early phase of COVID-19, baricitinib may prevent an excessive inflammatory response and the rapid progression of respiratory failure (74). Kurasawa et al. proposed that tofacitinib combined with conventional treatment might control refractory MDA5^+^ DM complicated with ILD (75). Furthermore, tofacitinib improved the survival 6 months after the onset of anti-MDA5+ amyopathic DM-associated ILD (76). In a randomized controlled trial, tofacitinib reduced the incidences of mortality and respiratory failure in patients with COVID-19 (77). Baricitinib and tofacitinib can cause thrombosis, and patients with COVID-19 are in hypercoagulable states; therefore, careful monitoring for the development of thrombosis during treatment with JAK inhibitors is necessary (78). JAK inhibitors, such as baricitinib and tofacitinib could be available treatments for COVID-19, thrombosis should be paid extensive attention to.

### Intravenous Immunoglobulin and Plasmapheresis

Intravenous immunoglobulin (IVIG) is effective in the treatment of refractory DM (79). IVIG improves patient strength, reduces mortality, and promotes recovery. There is a potential for patients with COVID-19 to respond to early immunotherapy, especially IVIG, which may also provide a variety of potential protective antibodies and anti-cytokine effects (80). Elevated cytokine levels are found in the sera of DM patients with ILD, and plasmapheresis is an option to remove the elevated cytokines as an additional supportive treatment (27, 67). Immunoglobulin and plasmapheresis might be considered in patients affected with SARS-CoV-2. A retrospective study demonstrated that COVID-19 patients who were not treated with DMARDs had a higher mortality rate than those who received methotrexate monotherapy, while leflunomide, antimalarials, TNF inhibitors, abatacept, belimumab, and IL-6 inhibitors were not associated with an increased risk of death (55). The efficacy of high-dose corticosteroids, IVIG, JAK inhibitors, and T-cell modulators for COVID-19 has been reported in a small series of cases, and clinical trials are currently under investigation (81).

## Conclusion and Perspectives

During the recent pandemic, several cases of COVID-19 were reported to be related to DM. In this review article, we discussed the possible relationship between COVID-19 and DM based on the similar potential pathogenic mechanisms and clinical manifestations. More importantly, we provide a direction for the treatment of COVID-19 through an analysis of immunosuppressive agents that are commonly used for DM. We have highlighted that the manifestations of DM, such as ILD and myositis, can also be observed in patients with COVID-19. Therefore, these manifestations of DM are difficult to distinguish in the early clinical stages, and even certain subtypes of DM my involve the same immune response that is caused by COVID-19. It is noteworthy that women have been more susceptible to DM during the COVID-19 pandemic and have more severe clinical symptoms, which means that extensive clinical attention should be given to the possibility of autoimmune diseases in female patients with COVID-19 and that the treatment regimens should be more aggressive in these patients.

In conclusion, infection with SARS-CoV-2 can induce the occurrence of DM. COVID-19 and DM potentially have a common pathogenesis, such as IFN1, which not only participates in organ damage of COVID-19 but also mediates muscle fiber damage in patients with DM. Although there have been some studies supporting the relationship between COVID-19 and DM, the exact mechanism is largely unknown, and more evidence is needed to confirm this association. Routinely available drugs for DM have provided an alternative therapeutic strategy for COVID-19. Further studies should be done to assess the efficacy and safety of the regimen to tackle the inflammatory stages of COVID-19. We recommend that all newly diagnosed DM cases should be tested for COVID-19 during the pandemic, especially for certain subtypes of DM patients, such as anti-MDA5+, and early differential diagnosis is helpful to improve the survival of these patients.

## Author Contributions

JQ: study design, data analysis, and manuscript revision. HX: literature search, data collection, and article writing. All authors contributed to the article and approved the submitted version.

## Funding

This project was sponsored by grants from China International Medical Foundation (Z-2018-40), Nantong Science and Technology Bureau Project (MSZ18108 and JCZ20058), and the Special Clinical Basic Research Key Project of Nantong University (2019JZ001).

## Conflict of Interest

The authors declare that the research was conducted in the absence of any commercial or financial relationships that could be construed as a potential conflict of interest.

## Publisher’s Note

All claims expressed in this article are solely those of the authors and do not necessarily represent those of their affiliated organizations, or those of the publisher, the editors and the reviewers. Any product that may be evaluated in this article, or claim that may be made by its manufacturer, is not guaranteed or endorsed by the publisher.
